# A customized regimen of intravitreal aflibercept for the treatment of choroidal neovascularization secondary to different chorioretinal diseases

**DOI:** 10.1186/s40942-023-00440-5

**Published:** 2023-01-18

**Authors:** Magdy Moussa, Mahmoud Leila, Mayada Ali Mohamed, Ahmed Osama Hashem

**Affiliations:** 1 Ophthalmology department, Faculty of Medicine, Tanta University, El Bahr Street, Tanta Qism 2, Gharbia Governorate, Tanta, 31111 Egypt; 2 Retina department, Research Institute of Ophthalmology (RIO), Giza, Egypt; 3 Ophthalmology department, Faculty of Medicine, Kafr El Sheikh University, Kafr El Sheikh, Egypt

**Keywords:** Intravitreal aflibercept, Secondary CNV, Customized IVA regimen, CNV secondary to chorioretinal diseases

## Abstract

**Background:**

To assess the response of CNV secondary to chorioretinal diseases to IVA and to explore the adequate dosing regimen and the long-term results.

**Methods:**

A retrospective study including patients with treatment-naïve active CNV secondary to chorioretinal diseases. All patients received an initial IVA injection followed by a PRN regimen. The main outcome measures were improvement of BCVA, improvement of anatomical morphology and vascularity of the CNV on SS-OCT, and SS-OCTA, respectively, and ocular or systemic complications attributed to IVA.

**Results:**

The study included 17 eyes of 15 patients. Nine patients (60%) were females. The median age was 20 years. The main primary chorioretinal disease was vitelliform macular dystrophy (29%). The mean baseline BCVA was 0.16. The mean follow-up period was 15 months. Final BCVA improved by a mean of 6 lines. The CNV regressed or became inactive in all eyes. The median number of IVA injections was 2. There were no ocular or systemic complications attributed to IVA.

**Conclusion:**

The customized IVA regimen is effective in inducing long-term regression of secondary CNV and in improving BCVA. Multimodal imaging is fundamental in establishing the diagnosis of CNV, and in monitoring its response to IVA.

## Background

The efficacy of intravitreal aflibercept (IVA) in treating neovascular age-related macular degeneration (nAMD) has been consolidated by landmark studies that demonstrated its non-inferiority to ranibizumab in terms of anatomical and visual outcomes [1–3]. The pharmacologic properties of aflibercept render it more avid than ranibizumab to vascular endothelial growth factor (VEGF) molecule. The resultant bondage with the VEGF molecule is stronger and more durable and enables instating a bi-monthly dosing regimen that is as effective as monthly ranibizumab but with less burden on the patient due to fewer injections delivered, the less cumulative risk of injection-related adverse events, and better quality of life [1, 4, 5]. Despite encouraging reports on the efficacy of IVA in choroidal neovascularization (CNV) secondary to pathologies other than nAMD, several aspects remain unexplained. While increased VEGF production is the common route on which different CNV-inciting pathologies converge, the response to anti-VEGF agents by CNV secondary to trauma, inflammation, and hereditary and degenerative disorders is not uniform and suggests that the VEGF load involved is largely modulated by the self-limited nature of the inciting pathology in contrast to the perpetual natural history of AMD [6–8]. Consequently, there is no consensus on the optimum initial dosing regimen and consecutive protocols for continuing treatment. This study aims to assess the response of CNV secondary to different fundus pathologies other than AMD to IVA and to explore the adequate dosing regimen and results of long-term follow-up.

## Methods

This is a retrospective study including consecutive patients who received IVA for CNV secondary to different fundus pathologies between 2015 and 2021. The study was conducted in a retina tertiary center in Egypt. We diagnosed CNV based on clinical examination, fundus fluorescein angiography (FFA); Topcon TRC 50DX fundus camera (Topcon Corporation, Tokyo, Japan) whenever applicable, swept-source optical coherence tomography (SS-OCT); DRI OCT Triton machine version 10.11 (Topcon Corporation, Tokyo, Japan), and swept-source optical coherence tomography angiography (SS-OCTA) imaging; [SS-OCTangio; OCTARA (Optical Coherence Tomography Angiography Ratio Analysis); Topcon Corporation, Tokyo, Japan]. Inclusion criteria included treatment-naïve active CNV. The criteria for diagnosis of CNV were symptoms of recent onset of diminution of vision and/or metamorphopsia, an elevated submacular lesion associated with subretinal hemorrhage (SRH), subretinal fluid (SRF), or retinal pigment epithelial detachment (PED) on biomicroscopy, a submacular early hyperfluorescence with progressive leakage on FFA, a fusiform submacular hyperreflective amorphous lesion with an overlying SRF on SS-OCT, and a hyperintense signal in the outer retinal layer or in the choriocapillaris with extensive arborization, anastomosis, and looping on SS-OCTA. The study excluded all patients with concomitant factors or retinal diseases that would confound the anatomical or visual outcome of IVA. These included patients with best-corrected visual acuity (BCVA)  ≤ counting fingers (CF) 30 cm, patients with previously treated CNV secondary to any fundus disease, patients with AMD, patients with axial length  ≥ 26 mm, active intra-ocular inflammation, diabetic maculopathy, retinal vascular occlusion, or optic nerve disease and patients with significant media opacity that would hinder sufficient image quality. We used a customized IVA injection regimen (Aflibercept 2.0 mg [EYLEA^®^, Regeneron Pharmaceutical Inc, Tarrytown, NY, USA)], per the fore-mentioned criteria of active CNV. The regimen consisted of a single injection without a prior loading dose followed by subsequent injections given pro re nata (PRN) if any or all of the following criteria were present: Newly developed SRH, persistent or increased intra-retinal fluid, or SRF  ≥ 100 µm on SS-OCT, decreased BCVA by  ≥ 1 line of vision, increased surface area, vascular arborization or flow density on SS-OCTA image of the outer retina or the choriocapillaris. If we detected CNV reactivation, we administered IVA injections monthly until the patient had complete regression of the CNV. The minimum period between 2 successive injections was 1 month. If there was no CNV activity, we followed up with the patient monthly. The main outcome measures were improvement of BCVA, improvement of anatomical morphology of the CNV in terms of reduction/disappearance of SRF with the improvement of retinal contour on SS-OCT, reduction/disappearance of the hyperintense signal on SS-OCTA, and ocular or systemic complications attributed to IVA.

### Statistical analysis

Data were collected, revised, coded, and entered into the Statistical Package for Social Science (IBM SPSS) version 23. The quantitative data were presented as mean, standard deviations, and ranges when parametric and median, and inter-quartile range (IQR) when data was found non-parametric. In addition, qualitative variables were presented as numbers and percentages. The comparison between two independent groups with quantitative data and nonparametric distribution was done by using the Mann-Whitney test. The comparison between two paired groups regarding quantitative data and nonparametric distribution was done by using the Wilcoxon Rank test. Spearman correlation coefficients were used to assess the correlation between two quantitative parameters in the same group. The confidence interval was set to 95% and the margin of error accepted was set to 5%. Thus, the p-value was considered significant at a level of P-value < 0.05.

## Results

The study included 17 eyes of 15 patients. Nine patients (60%) were females. The median age was 20 years (IQR 11–35; range 8–70). Five eyes (29%) had CNV secondary to vitelliform macular dystrophy. CNV was secondary to angioid streaks associated with pseudoxanthoma elasticum (PXE), multifocal choroiditis (MFC), punctate inner choroidopathy (PIC), and laser pointer injury in 2 eyes (12%) each. CNV was secondary to macular telangiectasia (MacTel) type II, Stargardt disease, Sorsby macular dystrophy, and juvenile X-liked foveoschisis in one eye (6%) each. The mean baseline BCVA was 0.16 decimal units (range 0.1–0.32; SD 0.08). The mean follow-up period was 15 months (range 6–48 months; SD 12). The mean final BCVA was 0.6 decimal units (range 0.32–1; SD 0.2), p < 0.001. Final BCVA improved by a mean of 6 lines. Thirteen eyes (76%) had a final BCVA  ≥ 0.5 decimal units. The CNV in all eyes either regressed completely or became inactive with a characteristic dead-tree appearance on SS-OCTA. The median number of IVA injections given was 2 (IQR 1–3; range 1–7). We did not detect ocular or systemic complications attributed to IVA injection Tables 1 and 2.

**Table 1:** Baseline characteristics of the study population

Baseline Characteristics	N (%)
Male	6 (40)
Female	9 (60)
Age, years	
< 10	2 (13)
10–30	8 (53)
30–50	4 (27)
> 50	1 (7)
Pathology	
Vitelliform macular dystrophy	5 (29)
PXE	2 (12)
MFC	2 (12)
Laser pointer injury	2 (12)
PIC	2 (12)
MacTel type II	1 (6)
Stargardt disease	1 (6)
Sorsby macular dystrophy	1 (6)
Juvenile X-linked foveoschisis	1 (6)
Mean baseline BCVA (decimal)	
0.1	10 (59)
> 0.1–0.32	7 (41)
Number of IVA injections	
1	6 (35)
2–5	9 (53)
> 5	2 (12)
Follow-up period (months)	
< 12	10 (59)
12–24	4 (23.5)
> 24	3 (18)

BCVA best-corrected visual acuity, IVA intravitreal aflibercept, MacTel macular telangiectasia, MFC multifocal choroiditis, PIC punctate inner choroidopathy, PXE pseudoxanthoma elasticum

**Table 2:** Difference between baseline and final BCVA

	Baseline	Final	Difference	Test value	P-value
BCVA					
Mean ± SD	0.16 ± 0.08	0.60 ± 0.23	0.44 ± 0.19	− 3.633 ≠	< 0.001
Range	0.1–0.32	0.32–1	0.22–0.7		
Range	213–660	208–619	− 370–309		

BCVA best-corrected visual acuity, SD standard deviation

### Case presentation

#### Case no. 1

**Fig. 1:**
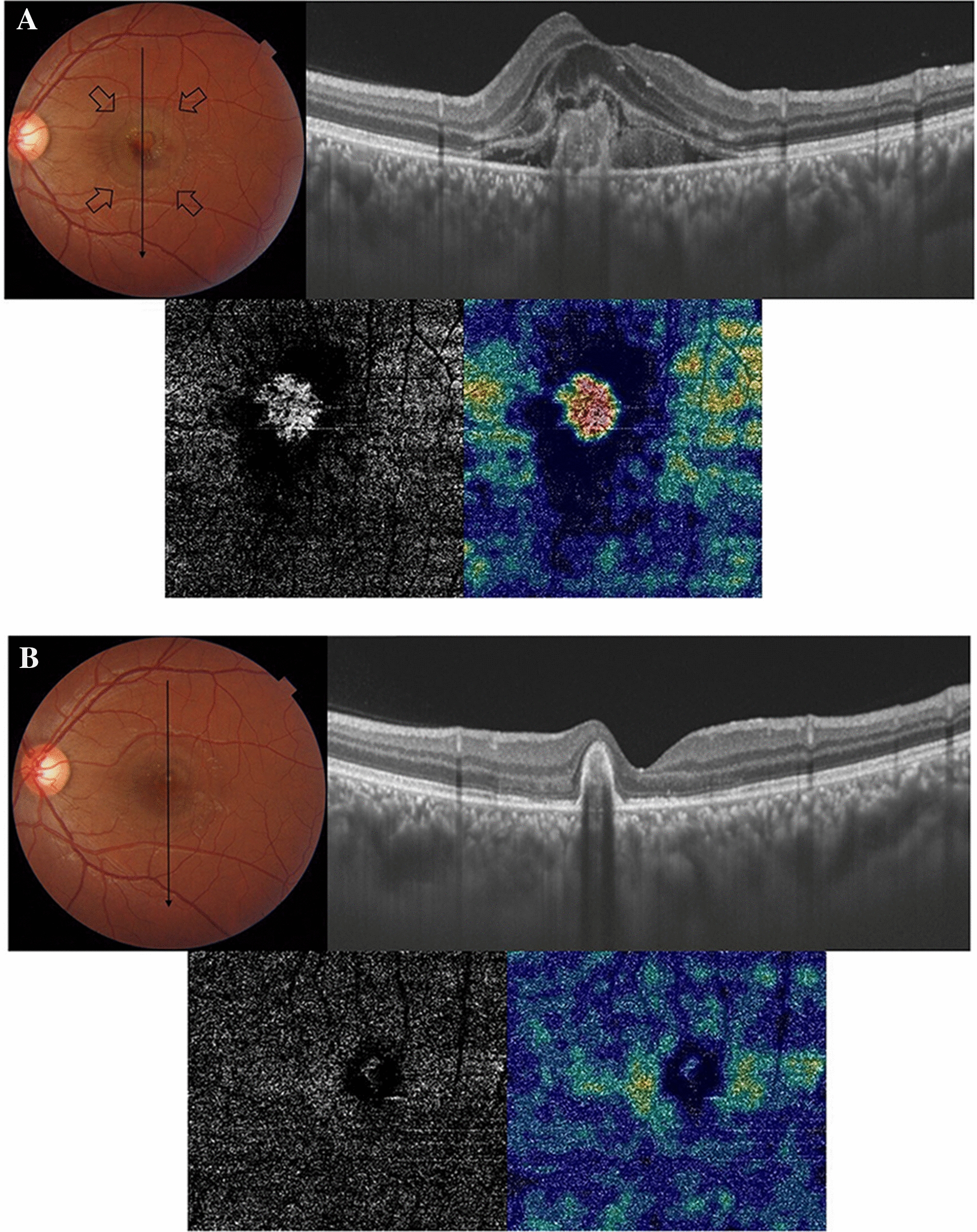
**A** Top left. Color fundus photo of the left eye of a 19-year-old male following laser pointer injury to the macular area. The macular area shows a well-circumscribed area of neurosensory detachment approximately 2-disc diameter in size (arrows) with central SMH and hard exudates. Top right. SS-OCT of the same eye in a line scan mode shows a sub-foveal amorphous hyperreflective lesion above the RPE. Note the overlying hyporeflective cystic space formation and multiple hyperreflective foci. Bottom. SS-OCTA image in a 3 × 3 mm field of the outer retina and the corresponding flow density map. Note the hyperintense signal with dense arborization, vascular anastomosis, and looping that are characteristic of an active neovascular network. Note the high flow within the neovascular network in the flow density image. **B** Top left. Color fundus photo of the same eye 6 months after a single IVA injection. The macular area shows resolution of neurosensory detachment, and SMH, which are replaced by a well-circumscribed submacular yellowish elevated lesion consistent with scar formation. Top right. SS-OCT in a line scan mode shows a parafoveal dome-shaped hyperreflective lesion above the RPE with the resolution of the previously noted cystic spaces. Bottom. SS-OCTA image in a 3 × 3 mm field of the outer retina and the corresponding flow density map. Note the resolution of the previously noted hyperintense signal of the neovascular network and the disappearance of high flow in the flow density map

A 19-year-old male patient presented to our clinic complaining of a marked drop of vision in his left eye following gazing into a laser pointer. His BCVA was 0.1. Fundus examination revealed a large neurosensory detachment in the macula with submacular hemorrhage (SMH) and hard exudates. SS-OCT revealed a sub-foveal hyperreflective amorphous lesion above the retinal pigment epithelium (RPE) consistent with SMH seen in the color photo. The lesion had an overlying SRF. SS-OCTA of the outer retina revealed a hyperintense signal characteristic of an active neovascular network. The patient received a single IVA injection. SS-OCT post-injection revealed restoration of the foveal contour with persistent organized SRH. SS-OCTA revealed a complete resolution of the previously noted hyperintense signal. His BCVA improved to 0.8. The patient did not have recurrence throughout 6 months of follow-up Fig. 1.

#### Case no. 2

**Fig. 2:**
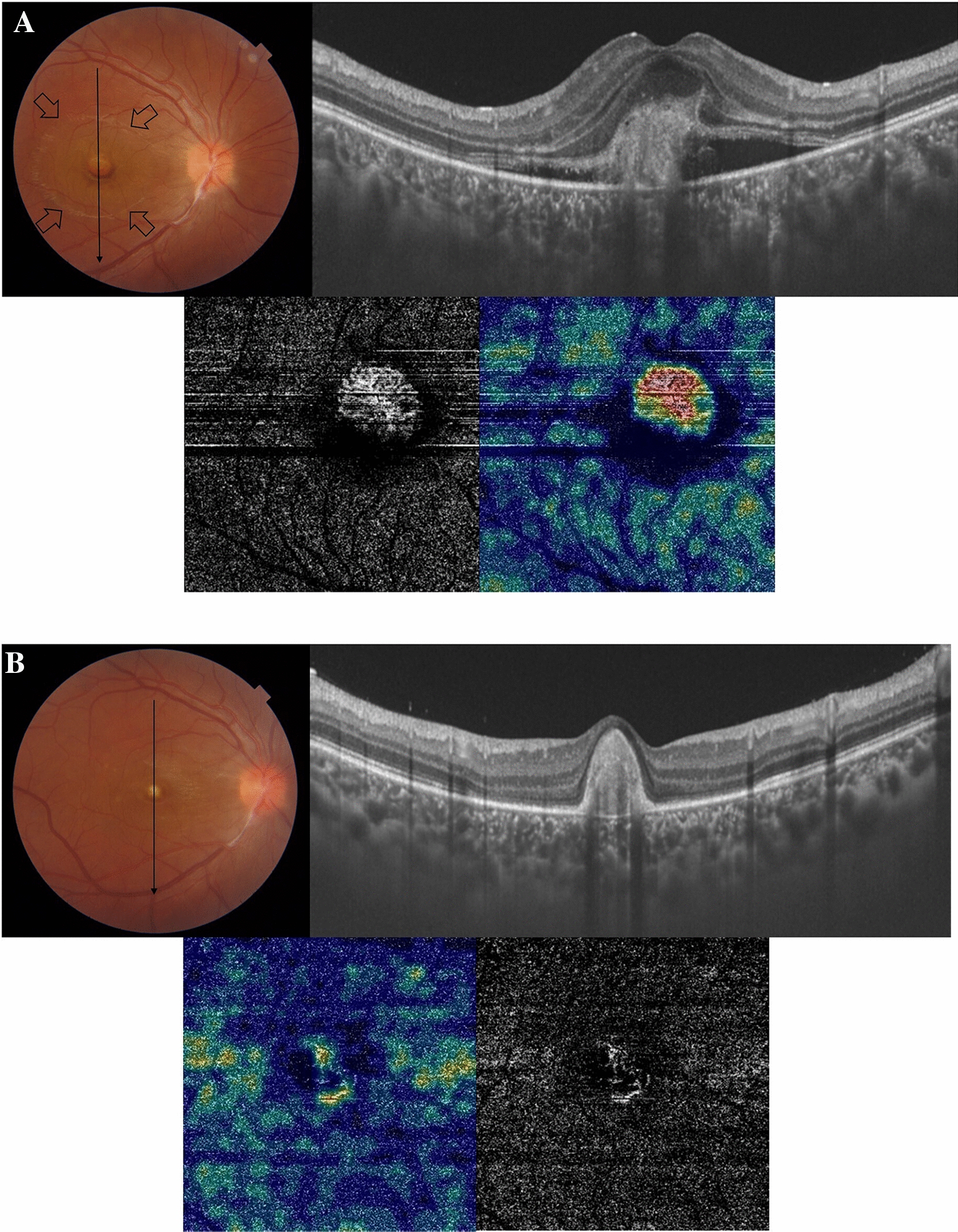
**A** Top left. The color fundus photo of the right eye of a 16-year-old female with a history of MFC. The macular area shows a well-circumscribed area of neurosensory detachment approximately 2-disc diameter in size (arrows) with a central subretinal yellowish-white lesion and a meniscus of SRH. Top right. SS-OCT of the same eye in a line scan mode shows diffuse macular thickening (spongy edema), multiple intra-retinal hyperreflective foci, a sub-foveal amorphous hyperreflective lesion above the RPE with surrounding neurosensory detachment and overlying turbid SRF. Bottom. SS-OCTA image in a 3 × 3 mm field of the outer retina and the corresponding flow density map. Note the hyperintense signal with dense arborization, vascular anastomosis, and looping that are characteristic of a large active neovascular network. Note the high flow within the neovascular network in the flow density map. **B** Top left. The color fundus photo of the same eye 6 months after 3 IVA injections. The macular area shows resolution of neurosensory detachment, and SMH, which are replaced by a well-circumscribed submacular yellowish elevated lesion consistent with scar formation. Top right. SS-OCT in a line scan mode shows a subfoveal dome-shaped hyperreflective lesion above the RPE with the resolution of the previously noted spongy edema, neurosensory detachment, and SRF. Bottom. SS-OCTA image in a 3 × 3 mm field of the outer retina and the corresponding flow density map. Note the low-intensity signal within residual filament-like vascular channels, and significantly lower flow in the flow density map compared to the baseline

A 16-year-old female patient who is a known case of MFC presented to our clinic with complaints of decreased vision in the right eye. Her BCVA was 0.1. There were no signs of activity of MFC. Fundus examination revealed a submacular yellowish-white elevated lesion with a meniscus of SRH and a surrounding larger area of neurosensory detachment. SS-OCT of the right eye revealed diffuse macular thickening (spongy edema), a sub-foveal hyperreflective amorphous lesion, neurosensory detachment, and overlying SRF. SS-OCTA of the outer retina revealed a hyperintense signal characteristic of an active neovascular network. The patient received 3 consecutive IVA injections separated by 4 weeks. Eventually, SS-OCT revealed resolution of the previously noted spongy edema, neurosensory detachment, and SRF though with a persistent dome-shaped hyperreflective lesion that was consistent with macular scarring in the color photo. SS-OCTA revealed a marked reduction in the size and vascularity of the CNV. Her BCVA improved to 0.32 and remained stable throughout 6 months of follow-up after the last IVA injection Fig. 2.

#### Case no. 3

**Fig. 3:**
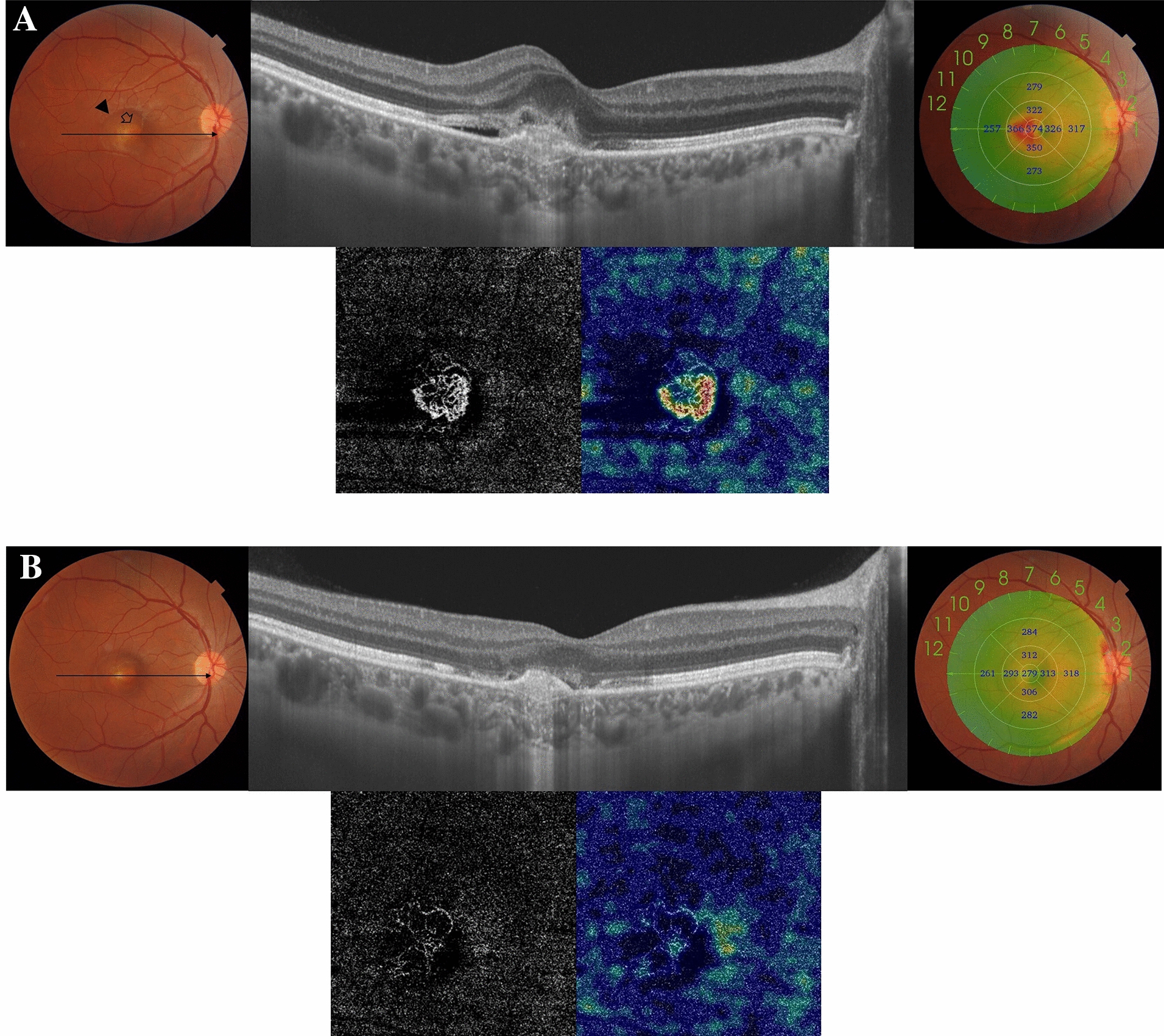
**A** Top left. The color fundus photo of the right eye of a 33-year-old female with a history of BVMD. The macular area shows the characteristic yellowish vitelliform lesion and scattered subretinal dot hemorrhages (arrow). Note the cuff of neurosensory detachment (black arrowhead). Top right. SS-OCT in a line scan mode shows a subfoveal hyperreflective vitelliform lesion with surrounding neurosensory detachment and underlying irregular flat PED (double layer sign). CMT measures 374 µm. Bottom. SS-OCTA in a 3 × 3 mm field of the outer retina and the corresponding flow density map. Note the typical hyperintense signal of an active neovascular network that exhibits dense anastomosis and looping in a typical lacy-wheel pattern. **B** Top left. The color fundus photo 36 months after a single IVA injection. Note the resolution of the previously noted subretinal dot hemorrhages and neurosensory detachment. Top right. SS-OCT in a line scan mode of the same eye. The macular area shows resolution of neurosensory detachment and reduction in the size of the previously noted subfoveal vitelliform lesion. CMT measures 279 µm. Bottom. SS-OCTA image of the outer retina in a 3 × 3 mm field and the corresponding flow density map. Note the almost complete resolution of the previously noted CNV hyperintense signal with residual discrete vascular streaks and minimal blood flow

A 33-year-old female patient who is a known case of Best vitelliform macular dystrophy (BVMD) presented to our clinic with complaints of decreased vision in the right eye. Her BCVA was 0.32. Fundus examination revealed the characteristic vitelliform lesion with scattered subretinal dot hemorrhages. SS-OCT of the right eye revealed a submacular hyperreflective amorphous lesion consistent with the vitelliruptive stage of BVMD, SRF, and irregular PED. SS-OCTA of the outer retina revealed a hyperintense signal characteristic of an active neovascular network. The patient received a single IVA injection. SS-OCT revealed a resolution of the previously noted SRF, with a persistent hyperreflective lesion. SS-OCTA revealed a marked reduction in the size and vascularity of the CNV. Her BCVA improved to 1.0 and remained stable throughout approximately 3 years of follow-up Fig. 3.

#### Case no. 4

**Fig. 4:**
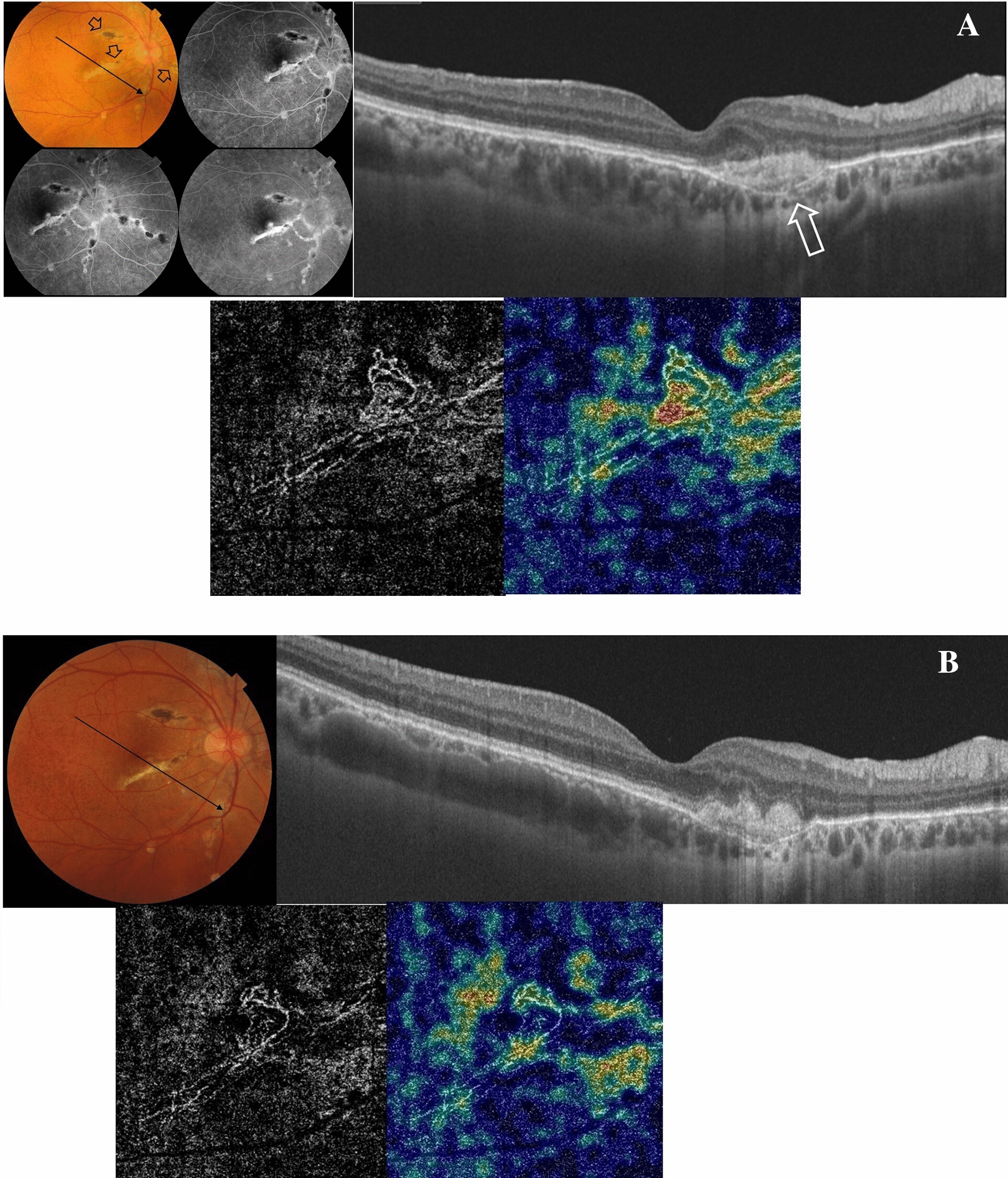
**A** Top left. The color fundus photo and FFA of the right eye of a 40-year-old female patient with angioid streaks and PXE. Note the linear streaks with RPE hyperpigmentation radiating centrifugally from the ONH margins (arrows). One of those streaks has bisected the fovea. Note the characteristic peau d’orange appearance of the fundus. FFA revealed transmission fluorescence of the streak bisecting the fovea. Note the subfoveal well-circumscribed mild but progressive leakage adjacent to the angioid streak that indicated a subfoveal CNV. Top right. SS-OCT of the macular area in a radial scan mode shows a subfoveal hyperreflective amorphous lesion with underlying conspicuous dehiscence of the RPE-Bruch’s complex (arrow) and choroidal excavation. Bottom. SS-OCTA image of the outer retina in a 3 × 3 mm field and the corresponding flow density map. Note the hyperintense signal of an active neovascular network with characteristic vascular looping and anastomosis and high flow density in the corresponding flow density map. **B** Top right. SS-OCT of the macular area in a radial scan mode 7 months after the patient received a single IVA injection. Note the reduction in the size of the previously noted subfoveal lesion. Bottom. SS-OCTA image of the outer retina in a 3 × 3 mm field and the corresponding flow density map. Note signs of inactivity in the form of the marked reduction of the previously noted hyperintense signal and disappearance of anastomosis and looping with the development of linear vascular streaks (dead-tree) and the low vascular flow in the flow density map

A 40-year-old female patient who is a known case of angioid streaks and PXE presented to our clinic with complaints of decreased vision in the right eye. Her BCVA was 0.32. FFA revealed a sub-foveal CNV adjacent to an angioid streak bisecting the fovea. SS-OCT of the right eye revealed a submacular hyperreflective amorphous lesion above the RPE. SS-OCTA of the outer retina revealed a hyperintense signal characteristic of an active neovascular network. The patient received a single IVA injection. SS-OCT revealed shrinkage of the previously noted hyperreflective lesion. SS-OCTA revealed a hyperintense signal of a residual linear vascular streak with a low vascular flow. Her BCVA improved to 1.0 and remained stable throughout 11 months of follow-up Fig. 4.

#### Case no. 5

**Fig. 5:**
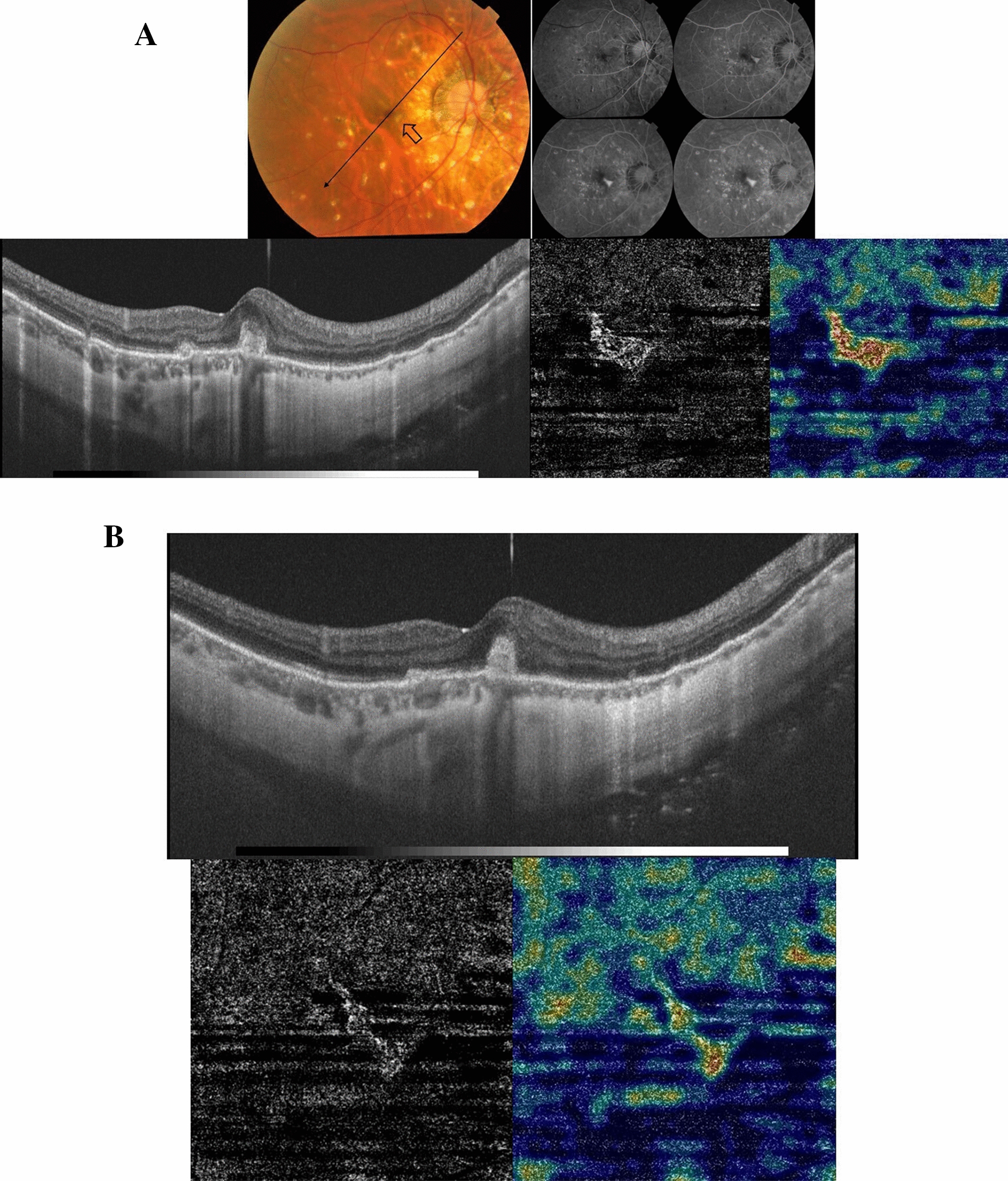
**A** Top. The color fundus photo and FFA of the right eye of a 37-year-old female patient with PIC and subfoveal CNV. Note the yellowish atrophic scars characteristic of healed PIC lesions in the posterior pole. The nasal juxtafoveal area shows a subretinal greenish elevated lesion (arrow). FFA revealed transmission fluorescence of the atrophic PIC lesions and early hyperfluorescence that increases in size and intensity denoting leakage from the subfoveal CNV and late staining of the same. Bottom. SS-OCT of the macular area in a line scan mode shows a subfoveal elevated hyperreflective amorphous lesion above the RPE. SS-OCTA image of the outer retina in a 3 × 3 mm field and the corresponding flow density map show the hyperintense signal of an active neovascular network and high flow density in the corresponding flow density map. **B** SS-OCT of the macular area in a radial scan mode and SS-OCTA image after 2 IVA injections. SS-OCT shows a reduction in the size of the previously noted subfoveal lesion. Note the reduction of the previously noted hyperintense signal with a low vascular flow in the flow density map on SS-OCTA

A 37-year-old female patient presented to our clinic with complaints of decreased vision in the right eye for 1 month. Her BCVA was 0.16 in the right eye and 1.0 in the left eye. Fundus examination revealed multiple well-circumscribed yellowish chorioretinal lesions localized to the posterior pole. There was no associated intraocular inflammation. She had a bilateral tilted disc and myopia of − 6 diopters. FFA of the right eye revealed the characteristic fluorescein pattern of atrophic PIC lesions along with a subfoveal CNV. SS-OCT revealed a subfoveal amorphous hyperreflective lesion overlying the RPE. SS-OCTA of the outer retina revealed a hyperintense signal characteristic of an active neovascular network. The patient received 2 IVA injections that induced regression of the CNV and the BCVA improved to 0.5. SS-OCTA revealed a significant reduction in the size and vascularity of the CNV. She had no recurrence of the CNV and the BCVA remained 0.5 throughout 18 months of follow-up Fig. 5.

## Discussion

In the present study, IVA induced regression of neovascular activity with an improvement of visual acuity in all 17 eyes, p < 0.001. Thirteen eyes (76%) achieved this outcome using  ≤ 3 IVA injections, of which 6 eyes (35%) required only a single injection. Several studies reported similar results for the use of IVA in cases of CNV secondary to trauma, laser-induced retinal injury, uveitis, and hereditary disorders. Diafas et al [9] reported a complete resolution of CNV secondary to laser pointer injury using a single IVA injection. They had no recurrences over 38 months of follow-up. Keles et al [10] reported a complete resolution of CNV secondary to alexandrite laser retinal injury using 3 IVA injections. They did not have recurrence over a 1-year follow-up. Another report by Arrevola et al [11] had regression of a CNV secondary to PIC after 2 IVA injections with no recurrences throughout 2 years of follow-up. Korol et al [12] administered a loading dose (3 consecutive injections) of IVA followed by a PRN regimen in a series of 15 eyes with a CNV secondary to toxoplasma chorioretinitis. They required a mean of 1.7 injections and they reported no persistent CNV during a 1-year follow-up. The authors did not detect serious ocular or systemic side effects. Gliem et al [13] reported a case series of 15 eyes with a CNV secondary to angioid streaks due to PXE. The treatment protocol included a single injection of IVA at the outset of enrollment followed by a PRN regimen. The authors administered a mean number of 6.7 injections over 1 year. They reported stable visual acuity in 67% of eyes. At the end of the follow-up period, 33% of eyes had persistent or recurrent CNV. None of the patients enrolled in the study had serious side effects related to IVA. The INTUITION study [8] reported a series of 16 patients with idiopathic CNV. The treatment protocol included a loading dose of 3 IVA injections followed by a treat-and-extend (TER) regimen up to week 20. Thereafter, a PRN protocol was used up to week 52. The authors reported significant improvement in BCVA, reduction in central retinal thickness, and CNV area. The mean number of IVA injections at the end of the follow-up was 5.4. There were no serious side effects related to IVA injection. In congruence with the safety reports of IVA by Korol et al [12], Gliem et al [13], and the INTUITION study [8], we did not detect any ocular or systemic side effects associated with IVA injection in the present study. Our results corroborate those of the fore-mentioned studies and support the notion that the damage induced to the RPE-Bruch’s membrane complex by certain fundus disorders with secondary CNV formation is self-limiting compared to the chronic progressive pathology of nAMD [14]. The reason could be that the primary triggering factor has ceased to exist after producing localized damage to the RPE-Bruch’s complex as in the case of trauma, laser injury, or resolved inflammation. Similarly, the damage sustained by the RPE-Bruch’s complex could be subtle and very slowly progressing as in hereditary diseases. Whatever the reason, the VEGF load produced is possibly far less than that produced secondary to AMD, where the progressive compromise of the RPE-Bruch’s membrane complex by drusen with consequent release of proangiogenic and proinflammatory cytokines leads to a perpetual release of VEGF and other angiogenic factors [14]. Therefore, it is prudent in the management of those patients to follow a reactive anti-VEGF injection protocol that is individualized for each patient per the response of the CNV to injection to avoid overtreatment and to reduce the risks associated with intraocular injections. In this sense, IVA could be a better agent than bevacizumab and ranibizumab due to its stronger bondage to VEGF and thus a longer duration of action [1, 4, 5]. An important point of strength in the present study is that we used a multimodal imaging protocol that included a tandem of FFA, SS-OCT, and SS-OCTA in the diagnosis and the follow-up of the response of CNV to IVA. The rationale for using this multimodal protocol is that the classic features that are pathognomonic of CNV on FFA and OCT are either displayed in an atypical manner or overlap with the signs of the primary disease in cases of secondary CNV. For instance, the vitelliform material in BVMD has the same optical properties as the fibrovascular tissue of CNV on OCT. Similarly, leakage on FFA is a common feature between inflammatory chorioretinal diseases and CNV. Consequently, these pathologies could be indistinguishable when using conventional FFA and OCT. In this sense, SS-OCTA is decisive in establishing the presence of CNV by displaying the hyperintense signal that is characteristic of high flow within a neovascular complex in the outer retina or the choriocapillaris [15–17]. The limitations of this study include its limited sample size and inhomogeneity in terms of the pathologies included. However, both factors might have been dictated by the relatively low prevalence of CNV secondary to the pathologies discussed.

## Conclusion

The customized IVA injection regimen is effective in inducing long-term regression of CNV secondary to trauma, laser maculopathy, uveitis, and hereditary disorders and in improving visual function. This approach avoids overtreatment and has a high safety profile. Multimodal imaging is fundamental in establishing the diagnosis of CNV. SS-OCTA is conclusive in the early detection of subtle CNV and in monitoring regression of the CNV during follow-up, particularly whenever OCT and FFA reveal equivocal results.

## Data Availability

All data pertaining to the present study are confidential. Access to these data will be granted exclusively to people or entities who meet the criteria for access to confidential data and only upon written request. All requests should be addressed to the corresponding author: Professor Magdy Moussa. Ophthalmology department, Faculty of Medicine, Tanta University. El Bahr street, Tanta Qism 2, Tanta, Gharbia Governorate, Egypt. Postal Code 31111.

## Abbreviations

BCVA: Best-corrected visual acuity
CF: Counting fingers
CMT: Central macular thickness
CNV: Choroidal neovascularization
DRI: Deep range imaging
FFA: Fundus fluorescein angiography
IQR: Interquartile range
IVA: Intravitreal aflibercept
MacTel: Macular telangiectasia
MFC: Multifocal choroiditis
µm: Micrometer
Mm: Millimeter
nAMD: Neovascular age-related macular degeneration
OCTARA: Optical coherence tomography angiography ratio analysis
PED: Pigment epithelial detachment
PIC: Punctate inner choroidopathy
PRN: Pro re nata
PXE: Pseudoxanthoma elasticum
RPE: Retinal pigment epithelium
SD: Standard deviation
SMH: Submacular hemorrhage
SS-OCTA: Swept-source optical coherence tomography angiography
SS-OCT: Swept-source optical coherence tomography
SRF: Subretinal fluid
SRH: Subretinal hemorrhage
VEGF: Vascular endothelial growth factor
